# Anti-c-Met antibodies recognising a temperature sensitive epitope, inhibit cell growth

**DOI:** 10.18632/oncotarget.1075

**Published:** 2013-06-29

**Authors:** Julin S. Wong, Emma Warbrick, Borek Vojtesek, Jeffrey Hill, David P. Lane

**Affiliations:** ^1^ p53 Laboratory, 8A Biomedical Grove, Immunos #06-06, Singapore, Singapore; ^2^ Epithelial Genetics Group, Medical Sciences Institute, College of Life Sciences and Medicine, Dentistry & Nursing, Dundee, Scotland, UK; ^3^ Regional Centre for Applied Molecular Oncology, Masaryk Memorial Cancer Institute, Brno, Czech Republic; ^4^ Experimental Therapeutics Centre, 31 Biopolis Way, Nanos Level 3, Singapore

**Keywords:** c-Met, seeMet 2, monoclonal antibody, temperature sensitive & cryptic epitope

## Abstract

c-Met is a tyrosine receptor kinase which is activated by its ligand, the hepatocyte growth factor. Activation of c-Met leads to a wide spectrum of biological activities such as motility, angiogenesis, morphogenesis, cell survival and cell regeneration. c-Met is abnormally activated in many tumour types. Aberrant c-Met activation was found to induce tumour development, tumour cell migration and invasion, and the worst and final step in cancer progression, metastasis. In addition, c-Met activation in cells was also shown to confer resistance to apoptosis induced by UV damage or chemotherapeutic drugs. This study describes the development of monoclonal antibodies against c-Met as therapeutic molecules in cancer treatment/diagnostics. A panel of c-Met monoclonal antibodies was developed and characterised by epitope mapping, Western blotting, immunoprecipitation, agonist/antagonist effect in cell scatter assays and for their ability to recognise native c-Met by flow cytometry. We refer to these antibodies as Specifically Engaging Extracellular c-Met (seeMet). seeMet 2 and 13 bound strongly to native c-Met in flow cytometry and reduced SNU-5 cell growth. Interestingly, seeMet 2 binding was strongly reduced at 4°C when compared to 37°C. Detail mapping of the seeMet 2 epitope indicated a cryptic binding site hidden within the c-Met α-chain.

## INTRODUCTION

c-Met is a 190 kD tyrosine kinase receptor made up of an extracellular α-chain which is linked by a disulphide bond to a transmembrane β-chain. c-Met is synthesised as a 170 kD single polypeptide that is proteolytically cleaved to form the α-chain and the β-chain [1]. The mature α-chain is 45 kD and constitutes part of the sema domain. The sema domain is a conserved domain shared by semaphorins and plexins. This domain adopts a seven-bladed beta-propeller structure which is important for homo-dimerisation. In c-Met, both the α-chain and the β-chain form the sema domain that is necessary and sufficient for receptor dimerisation and ligand binding [2]. The 140 kD mature β-chain consists of an extracellular domain, a transmembrane domain and a cytoplasmic domain. The extracellular portion of the β-chain makes up the remainder of the sema domain. The cytoplasmic portion of c-Met β-chain contains the juxtamembrane region followed by a kinase domain and a carboxyl-terminal tail. The carboxyl-terminal tail is essential for c-Met downstream signaling as it contains the docking site for signaling and adapter proteins that bind to c-Met.

Hepatocyte growth factor (HGF) is the only known c-Met ligand. Upon HGF binding, c-Met receptor dimerises on the cell surface which results in autophosphorylation of tyrosine residues (Y1230, Y1234 and Y1235) in the kinase domain. Autophosphorylation of Y1234 and Y1235 is thought to induce a conformational change in c-Met, exposing the docking site (Y^1349^VNVXXXY^1356^VHV) in the carboxyl-terminal tail of c-Met [3]. This results in transphosphorylation of tyrosine residues (Y1349 and Y1356) in the c-Met docking site. The docking site becomes available for recruitment of adaptor and signalling molecules resulting in the activation of various signalling pathways including the AKT/PI3K, RAS/MAPK and STAT pathways [3].

Aberrant c-Met activation of c-Met signalling pathways correlates with hyperproliferation, tumour cell invasion, tumour angiogenesis and poor prognosis in various human cancers. In addition, c-Met signalling protects the tumour cell by inhibiting apoptosis and inducing resistance towards cancer therapy, thus hampering the efforts of tumour treatment. c-Met as a cancer prognosis marker and its involvement in cancer metastasis and drug resistance makes c-Met a very attractive drug target. Many antibodies targeting tyrosine kinase receptors such as Herceptin (clinically known as Trastuzamab), have been successful in the clinic. Herceptin is a chimeric antibody targeted against the tyrosine receptor kinase HER2, used for breast cancer treatment. With the success of therapeutic antibodies, attempts have been made to develop therapeutic antibodies against the Met-HGF axis. Neutralising antibodies targeted against HGF aimed to block Met-HGF interaction were developed. c-Met binding to HGF was only blocked when a combination of three different anti-HGF antibodies were used [4]. Similarly, van der Horst *et al.* [5] reported the combination of using two fully human anti-Met antibodies (R13 and R28) was more effective in inhibiting c-Met binding to HGF as compared to using R13 or R28 alone. Burgess *et al.* [6] developed five fully human anti-HGF antibodies targeted against the β-chain of HGF. These antibodies were successful in blocking Met-HGF interaction in U-87MG glioblastoma cells.

Developing therapeutic bivalent antibodies targeted against c-Met has been challenging. Prat *et al.* [7] developed two monoclonal antibodies (DO-24 and DN-30) against the extracellular domain of c-Met. Interestingly, both monoclonal antibodies act as an agonist rather than an antagonist and activate c-Met signaling *in vivo*. It is hypothesised that the bivalent binding of an antibody could mimic the dimerising effect of HGF, thus causing c-Met dimerisation upon antibody binding. To avoid c-Met activation by bivalent monoclonal antibodies, Pacchiana *et al.* [8] engineered the DN-30 Fab fragment. DN-30 Fab retained its high binding affinity towards c-Met but lost its agonist activity towards c-Met. DN-30 Fab efficiently inhibited c-Met signaling by causing c-Met ectodomain shedding and receptor down regulation [8]. The one-arm 5D5 antibody (MetMab or clinically known as Onartuzumab) is a monovalent chimeric antibody targeted against c-Met developed by Genetech [9]. Like DN-30, bivalent 5D5 antibody became an antagonist when converted to a monovalent Fab [10]. In contrast to Fab DN-30, MetMab acts as an antagonist by competing with HGF for c-Met binding and causes c-Met internalisation and down-regulation [10]. Recently, Greenall *et al.* [11] was the first to report bivalent anti-Met monoclonal antibodies that are not agonists. LMH 87 antibody, that targets the α-chain of c-Met, was shown to cause c-Met down-regulation by receptor internalisation.

This study describes the development of a panel of bivalent anti-Met murine monoclonal antibodies. These antibodies were raised against the α-chain of human c-Met and are termed Specifically Engaging Extracellular c-Met (seeMet). seeMet antibodies were characterised by Western blotting, immunoprecipitation, flow cytometry, epitope mapping and agonist/antagonist activity towards c-Met. Surprisingly, none of these antibodies were c-Met agonists. Two antibodies, seeMet 2 and 13, showed the strongest binding to native c-Met by flow cytometry but work poorly to detect denatured c-Met on Western blots. In contrast seeMet 11 and seeMet 12 antibodies showed outstanding specificity in Western blot analysis. seeMet 2 was the most effective in reducing cell division. Further analysis of seeMet 2 on flow cytometry showed that its binding to c-Met on live cells is temperature sensitive. Detailed mapping of seeMet 2 epitope revealed that part of seeMet 2 epitope is buried within the reported native crystal structure of c-Met.

## RESULTS

### Development and initial characterisation of seeMet antibodies

The α-chain of human c-Met was prokaryotically expressed and purified. Purified α-chain was used to immunise BALB/c mice. To obtain hybridoma cells producing anti-α-chain c-Met antibodies, the spleen cells of immunised mice were fused with SP2./0-Ag14 cells. Hybridoma cells were single-cell cloned and cell supernatant from monoclonal hybridoma clones were screened for anti-α-chain c-Met reactivity mainly by Western blotting and cell staining. Post primary and secondary antibody screening (Supplementary Figure 1), a panel of 21 seeMet antibodies were selected for isotype characterisation and epitope mapping. Antibody isotyping was performed by dipping commercially-available isotyping strips into monoclonal hybridoma supernatant. All 21 monoclonal antibodies share the same IgG isotype (but not the same subclass) and kappa light chain (Table 1).

**Table 1:** Epitope mapping and isotyping of seeMet monoclonal antibodies

Binding region	seeMet antibody	IgG subclass	Light chain	Sequence of binding region / mAb epitope mapping
1	16	IgG2A	Kappa	KVAEYKTGPV
2	12	IgG1	Kappa	LEHPDCFPCQDCSSK
	20			
3	11	IgG1	Kappa	CFPCQDCSSK
	21			
4	14	IgG1	Kappa	LVVDTYYDDQ
	18			
17
19	IgG2B
5	3	IgG1	Kappa	LISCGSVNRG
	5			
6	4	IgG2A	Kappa	LISCGSVNRGTCQRH
7	1	IgG1	Kappa	QIEEPSQCPD
	2			
9
8	6	IgG1	Kappa	FRDS
	10			
13
17
9	8	IgG1	Kappa	KETKDGFMFL
10	7	IgG1	Kappa	NSGLHSYMEM

**Figure 1:**
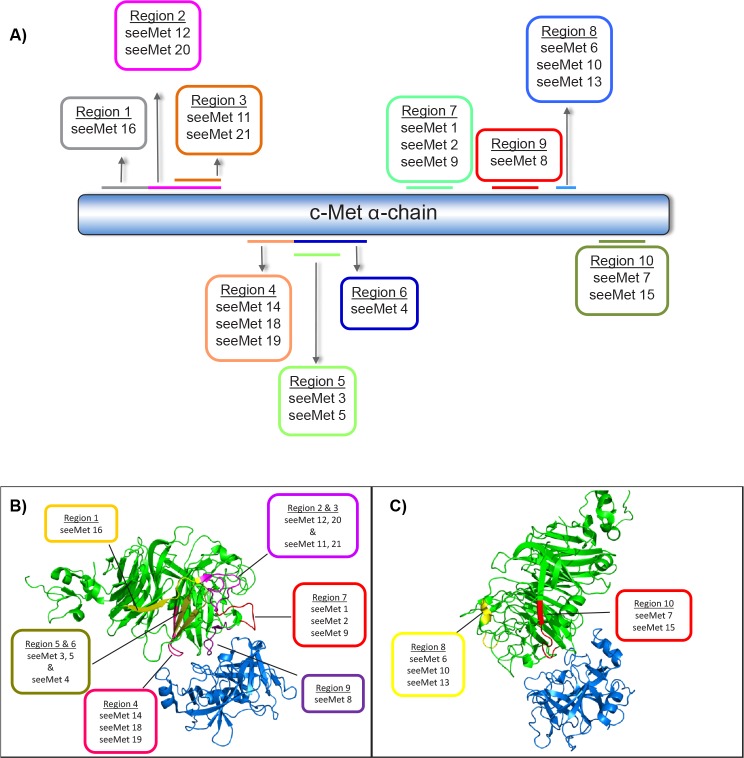
seeMet monoclonal antibody epitope mapping A) Schema of antibody binding regions. Pepscan, an ELISA-based assay, was used to determine the binding region of 21 monoclonal hybridoma supernatants. Consecutive overlapping peptides that span the entire c-Met α-chain were synthesised and coated in 96-wells. To determine the region of antibody binding on the α-chain, monoclonal cell supernatants were added to each peptide. Antibody binding results in a colourmetric reaction which is analysed by absorbance reading at 450 nM. The epitope of the antibodies were categorised into regions. Antibodies that share the same binding region are indicated. Figure not drawn to scale. B & C) Mapping of antibody binding regions to the crystal structure of c-Met extracellular domain (amino acid residues 25 – 567), binding to HGF β-chain. Crystal structure was obtained from Protein Data Bank (PDB), accession number 1SHY. c-Met is highlighted in green and HGF in blue. The protein complex is shown from two different viewpoints (B) and (C) to allow visualisation of the different antibody binding regions in relation to ligand-receptor interaction site.

Epitope mapping of the 21 seeMet monoclonal antibodies was determined by an ELISA-based assay (Pepscan). Consecutive, overlapping synthetic peptides that span the entire c-Met α-chain were biotinylated and added to strepatvidin-coated plates. Antibody was then added to the peptides and binding was determined colorimetrically. In total, 10 different antibody binding regions from the α-chain were identified from the 21 monoclonal cell supernatants tested, indicating that there is no one main region that is highly immunogenic. A simplified diagram of the antibody binding regions on c-Met α-chain is shown in Figure 1A. The antibody binding regions were also mapped onto the crystal structure of c-Met, PDB accession number 1SHY (Figure 1B & C). The results of initial antibody characterisation are summarised in Table 1.

### Further characterisation of purified seeMet monoclonal antibodies

From the panel of 21 seeMet monoclonal antibodies, 11 antibodies were selected for production and purification. Purified antibodies were further characterised by Western blotting, immunoprecipitation, flow cytometry, and agonist/antagonist activity by cell scattering.

seeMet monoclonal antibodies were purified using protein A beads. Purified antibody was resolved on a SDS-PAGE gel and stained with Coomassie blue dye (data not shown). Other than the expected two bands corresponding to the heavy (~51 kD) and light (~25 kD) chain of an antibody, no other protein bands were observed in the Coomassie-stained gel, suggesting that the purification of monoclonal antibodies was successful.

**Figure 2:**
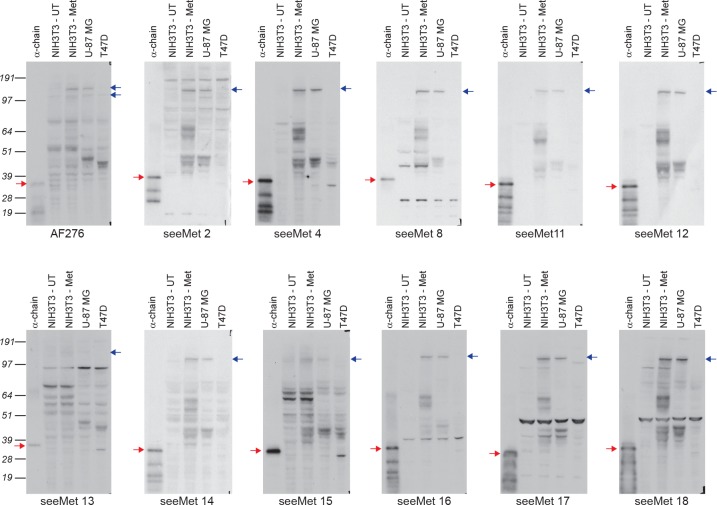
Purified seeMet monoclonal antibodies on Western blotting 11 hybridoma clones were selected for antibody production. Monoclonal antibodies were purified and tested again for reactivity against full length c-Met in whole cell lysates. 50 μg of whole cell lysate was obtained from untransfected NIH3T3 cells (UT), NIH3T3 cells transfected with full length human c-Met (Met), U-87 MG cell lines and T47D cell lines. U-87MG cells and T47D cells express high levels and non-detectable levels of c-Met respectively. Monoclonal antibodies are used at concentration of 1 μg/mL. 10 ng of purified α-chain was used as positive control. AF276 antibody was used as control. Blue arrows indicate c-Met precursor (170 kD). Red arrows indicate purified α-chain. Molecular weights are noted aside, in kilodaltons.

To ensure that the monoclonal antibodies retained their anti-α-chain activity after production and purification, purified monoclonal antibodies were characterised by Western blotting (Figure 2). 1 μg/mL of purified monoclonal antibodies was used to detect purified c-Met α-chain, c-Met expressed after transfection of NIH3T3 cells and endogenous c-Met in U-87MG cells. All monoclonal antibodies, except seeMet 13 and 15, successfully detected purified c-Met α-chain, and transfected as well as endogenous human c-Met. seeMet 13 and 15 had low specificity towards c-Met. seeMet 2 and 14 were observed to have higher affinity towards c-Met, compared to seeMet 13 and 15. seeMet 4, 8, 16, 17 and 18 have higher affinity towards c-Met and fewer non-specific bands compared to seeMet 2, 13, 14 and 15. Despite recognising different epitopes (Figure 1A), most of the seeMet monoclonal antibodies share the same pattern of band recognition with seeMet 11 and 12. This suggests that the band profiles of seeMet 11 and 12 are completely specific. seeMet 11 and 12 are thus the best monoclonal antibodies to use on Western blots as they detected c-Met with good affinity and specificity. Horseradish peroxidase (HRP) was conjugated to seeMet 12 (seeMet 12-HRP) and used as a tool in this study for detecting precursor c-Met and mature c-Met α-chain.

**Figure 3:**
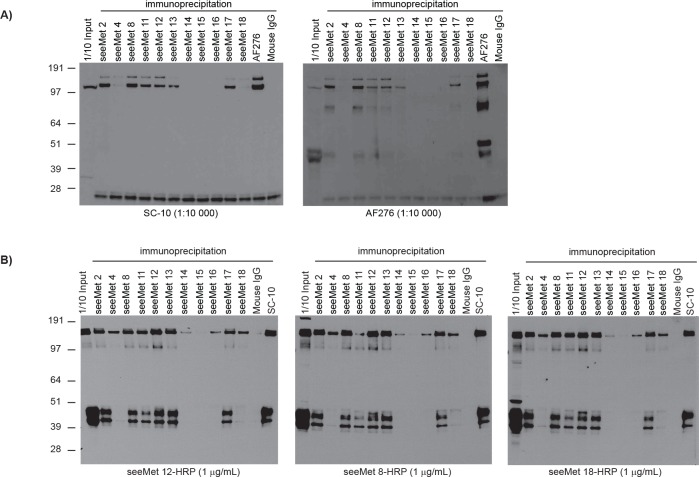
seeMet monoclonal antibodies immunoprecipitate endogenous c-Met Monoclonal antibodies were produced and purified using protein A beads. Purified antibodies were tested for their ability to immunoprecipitate endogenous c-Met from SNU-5 cell lysate. 1 µg/mL of antibodies were used for immunoprecipitation. Commercial anti-Met antibodies, SC-10 and AF276, and mouse IgG were used as controls. Immunoprecipitated SNU-5 cell lysate was analysed by Western blotting using A) SC-10 and AF276 antibody, and B) HRP-conjugated monoclonal antibodies developed from this screen. seeMet 8, 12 and 18 were previously shown to recognise different epitope on c-Met and work well on Western blots. Molecular weights are noted aside, in kilodaltons.

To further characterise the monoclonal antibodies reactivity against c-Met α-chain, the antibodies were used to immunoprecipitate endogenous c-Met from SNU-5 cell lysate. SNU-5 is a human gastric cell line that expresses high levels of c-Met. Immunoprecipitated cell lysates were analysed by Western blotting (Figure 3) using the commercial SC-10 and AF276 antibody. Although SC-10 and AF276 antibodies are raised against different regions of c-Met, both SC-10 and AF276 share similar Western blot band profiles. Most of our monoclonal antibodies successfully immunoprecipitated the 170 kD precursor c-Met (α- and β- chain linked together). Interestingly, a 145 kD protein band, which corresponds to the mature c-Met β-chain, was also observed on the Western blot, suggesting that the c-Met β-chain was pulled down together with its α-chain. In order to ensure that mature α-chain was immunoprecipitated, seeMet 12-HRP (developed from this screen) was used to analyse the same immunoprecipitated SNU-5 cell lysate on Western blots. seeMet 8 and 18, which was also developed in this screen, were conjugated to HRP and also used to detect c-Met α-chain. seeMet 8, 12 and 18 recognise different epitopes on c-Met (Figure 1A) but all successfully detected mature c-Met α-chain and precursor c-Met strongly (Figure 3).

**Figure 4:**
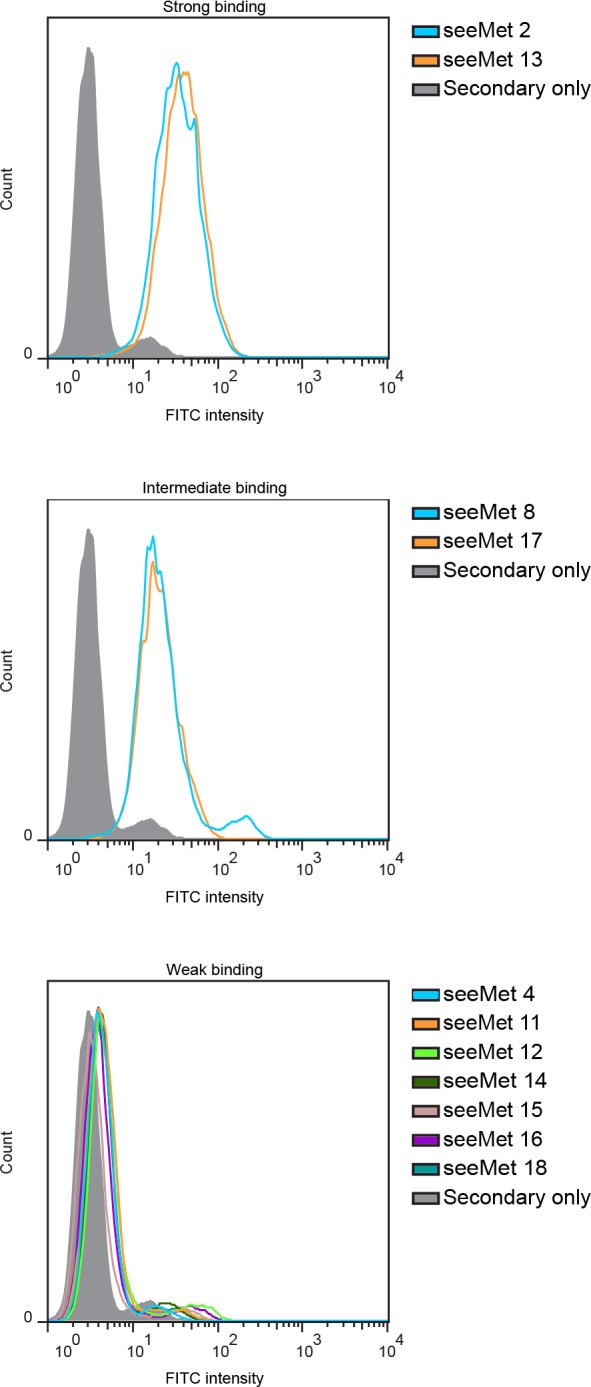
Flow cytometry analysis of SNU-5 cells treated with seeMet monoclonal antibodies Purified monoclonal antibodies (1 μg/mL) were incubated with live SNU-5 cells. Bound antibodies were detected using FITC-conjugated secondary antibodies before the cells were passed through a flow cytometer. A shift in FITC intensity indicates monoclonal antibodies binding to SNU-5 cells.

Flow cytometry was used to determine if our monoclonal antibodies could bind to native c-Met on live cells. SNU-5 cells were incubated with 1 µg/mL of monoclonal antibody. Binding of seeMet monoclonal antibodies to native c-Met expressed on the cell surface of SNU-5 cells was detected using anti-mouse secondary antibody conjugated with FITC dye. T47D, a human breast cancer cell line expressing low levels of c-Met, was used in this assay to determine if the antibodies bind non-specifically to the cell surface. The flow cytometry results of purified monoclonal antibody treated T47D cells were indistinguishable from the negative control (secondary antibody only), indicating that monoclonal antibodies do not bind non-specifically to the cell surface (data not shown). Flow cytometry results obtained with SNU-5 cells showed that the antibodies fell into three distinct groups with different fluorescence intensity towards native c-Met: strong fluorescence intensity, intermediate fluorescence intensity and weak fluorescence intensity (Figure 4). seeMet 2 and 13 showed the strongest fluorescence intensity in this assay. Surprisingly, these antibodies showed poor affinity for c-Met by Western blotting. seeMet 8 and 17 showed intermediate fluorescence intensity while seeMet 4, 11, 12, 14, 15, 16 and 18 showed weak fluorescence intensity (as demonstrated by the small peak shift).

**Figure 5:**
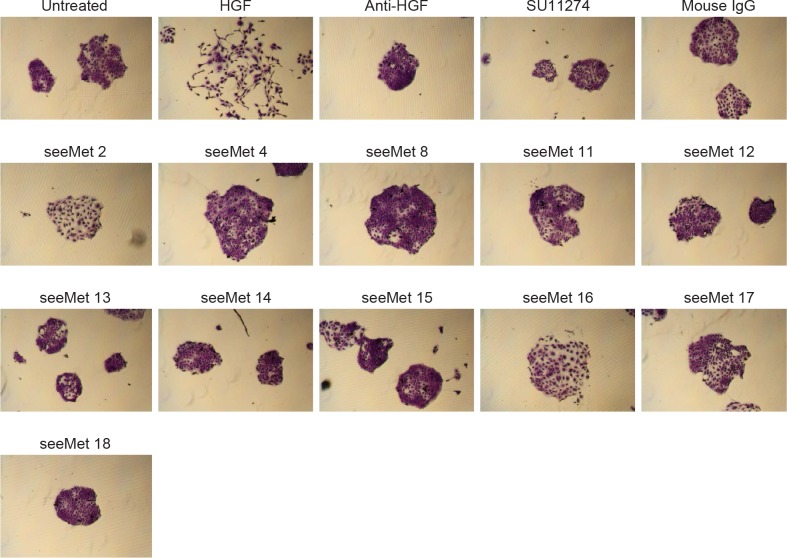
Effects of purified seeMet monoclonal antibodies on cell scatter HaCaT cells were serum-starved for 24 hrs. Monoclonal antibodies (1 μg/mL) and HGF were then added to the cells for a further 24 hrs before fixation. Cells were then stained with crystal violet for visualisation. Anti-HGF antibody (anti-HGF), commercially-obtained mouse immunoglobulin G (IgG), and c-Met small molecule inhibitor SU11274 (5 µM) were used as controls.

Cell scattering is one of the biological hallmarks of c-Met activation. Agonist bivalent monoclonal antibodies targeting c-Met would activate c-Met and cause cells to become motile and disperse. Here, we examine the effects of purified seeMet monoclonal antibodies on cell scatter. HaCaT cells were seeded at low density and allowed to grow until colonies formed. Cells were serum-starved for 24 hrs before incubating with 1 μg/mL of monoclonal antibodies for 24 hrs. Cells were then fixed and stained with crystal violet. As a control, cell scatter of HaCaT cells was induced with 10 ng/mL of HGF (Figure 5). The presence of non-specific IgG (mouse IgG) did not affect scattering. Anti-HGF antibody and SU11274 were used as controls. SU11274 is a small molecule inhibitor of c-Met. Anti-HGF and SU11274-treated cell colonies remained circular. No cell scatter was observed in cells treated with monoclonal antibodies alone (Figure 5). This suggests that the monoclonal antibodies are not agonist towards c-Met activated cell scatter.

### seeMet 2 and 13 reduce cell growth in SNU-5 cells

**Figure 6:**
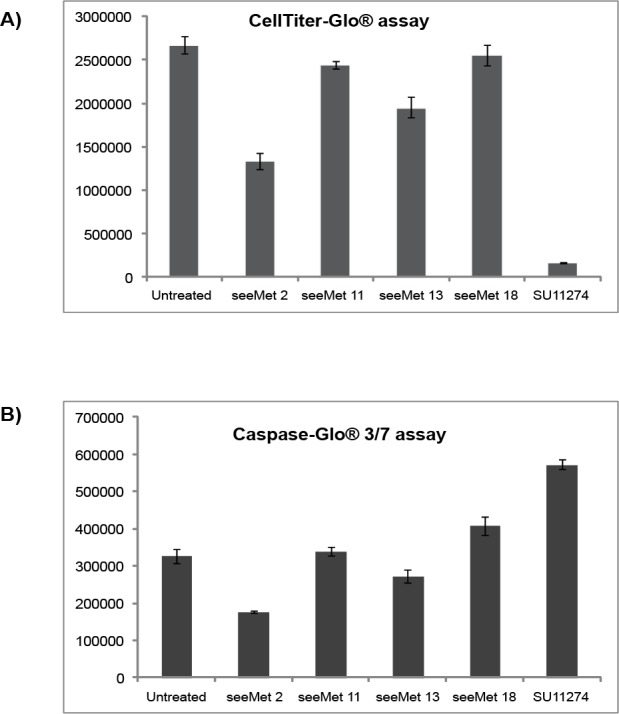
Effects of purified seeMet monoclonal antibodies on SNU-5 cell viability and caspase activation 10 µg/mL of monoclonal antibody were added to SNU-5 cells in the presence of serum containing media. Cells were incubated for 72 hrs before (A) cell viability, as judged by ATP levels in the CellTiter-Glo® assay, and (B) caspase activation were tested. SU11274 (5 µM) was used as control.

**Figure 7:**
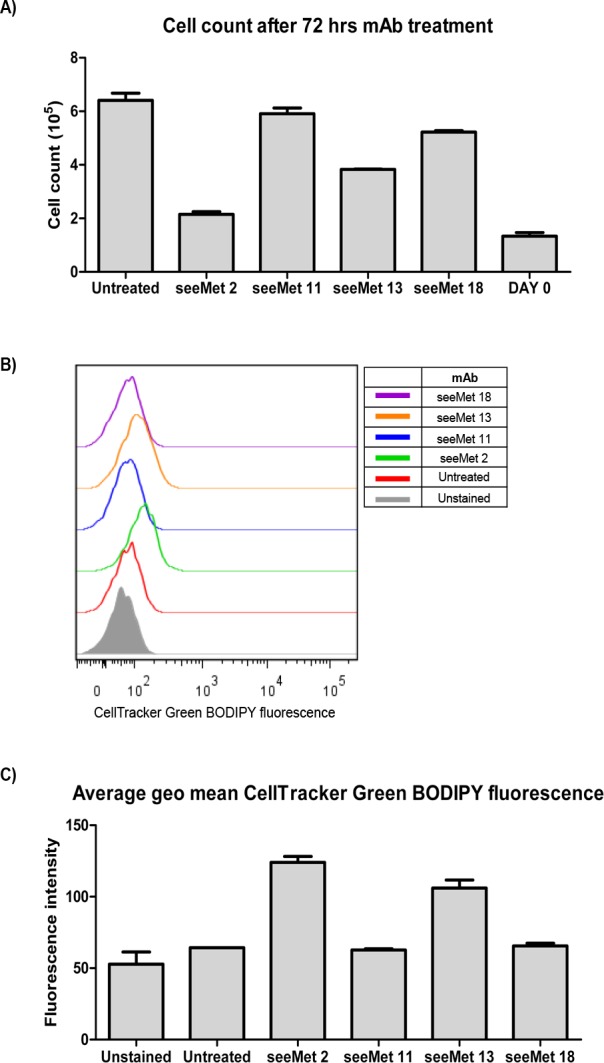
seeMet 2 and 13 inhibit cell growth 10 µg/mL of monoclonal antibody were incubated with SNU-5 cells in the presence of serum containing media. A) 72 hrs post monoclonal antibody treatment, cells were harvested and counted using the automated ADAM cell counter (Digital Bio). DAY 0: number of cell seeded for the assay. B & C) SNU-5 cells were stained with 5 µM CellTracker Green BODIPY dye before incubating with monoclonal antibody for 6 days. Fresh media containing 10 µg/mL of monoclonal antibody was added to the cells on alternate days. Cells were harvest and CellTracker Green BODIPY fluorescence retention was analysed by flow cytometry. B*)* Histogram representation of fluorescent cells treated with respective monoclonal antibody. C) CellTracker Green BODIPY fluorescence intensity of monoclonal antibody treated cells was recorded by flow cytometry. Experiment was performed in duplicates. Average geometric mean fluorescence was obtained and plotted.

seeMet 2 and 13 showed the strongest binding to native c-Met in flow cytometry. In order to determine if these antibodies have any physiological effects on tumour cells, 10 µg/mL of seeMet antibody was added to SNU-5 cells. The effects of seeMet 2 and 13 on SNU-5 cell viability and caspase activation were recorded after 72 hrs of antibody treatment (Figure 6). seeMet 11 and 18, which demonstrated weak affinity in flow cytometry, were used as controls. SU11274 showed a significant reduction in cell viability and high caspase activation. This indicates that SU11274 acts by induction of caspase activity which in turn result in apoptosis of SNU-5 tumour cells. Cells treated with seeMet 2 or 13 appear to have reduced cell viability in the CellTiter-Glo® assay, and reduced caspase activation in the Caspase-Glo® 3/7 assay. To determine the effects of seeMet monoclonal antibody on cell growth, a cell count was performed 72 hrs post antibody treatment. Remarkably, almost no increase in cell number was detected in cells exposed to seeMet 2 implying that seeMet 2 inhibited cell division completely. seeMet 2 showed the strongest cell growth reduction followed by seeMet 13. In this assay, seeMet 18 induced only a slight inhibition of cell division and seeMet 11 had no effect (Figure 7A). Blocking cell division would result in the apparent reduction of cell viability in the CellTiter-Glo® assay which measures ATP concentration and a reduction in the background levels of caspase activation in seeMet 2 and 13 treated cells.

To further demonstrate the effects of seeMet monoclonal antibody on cell growth, SNU-5 cells were pre-stained with the CellTracker green BODIPY dye before antibody treatment. CellTracker BODIPY dye is a membrane permeable dye that enters cells freely. Once in the cell, the dye is converted to a membrane impermeable product which labels the cell green. The dye is passed on to dividing daughter cells but is not transferable to neighbouring cells. Dividing cells would fail to retain the same intensity of green dye as it is being diluted to progeny cells. Cells were treated with 10 µg/mL of monoclonal antibodies for 6 days and dye retention in cells was analysed by flow cytometry. As expected, the levels of fluorescence intensity in untreated cells, seeMet 11 and seeMet 18 treated cells fell to levels similar to unstained cells (Figure 7B & C). seeMet 2 and 13 treated cells retained significant levels of green fluorescence indicating an absence of cell division.

### Internalisation of seeMet 2 and 13 in SNU-5 cells

**Figure 8:**
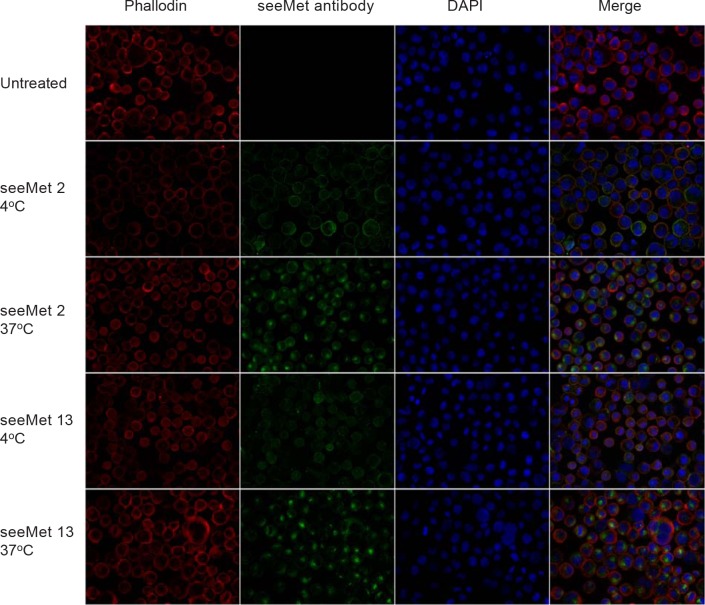
Intracellular immunofluorescence staining of seeMet 2 and 13 in SNU-5 cells Live SNU-5 cells were incubated with 10 µg/mL of monoclonal antibody for an hour at 4°C or 37°C. Cells were harvested and spun onto microscope slides. Cells were fixed in 4% paraformaldehyde and permeablised. seeMet 2 and 13 were detected using anti-mouse Alexa Fluor®488-conjugated secondary antibody. Phallodin and DAPI, which stained F-actin and nucleus respectively, were used as counter stain.

In order to determine if seeMet 2 and 13 were internalised into the cell upon antibody binding, SNU-5 cells were incubated with 10 µg/mL of monoclonal antibody. Monoclonal antibody treated cells were fixed and permeablised. Bound and internalised antibody was detected by anti-mouse Alexa Fluor®488-conjugated secondary antibody. Antibody localisation was observed by confocal microscopy. At 4°C, staining of both seeMet 2 and 13 were observed to be localised on the cell membrane with diffused cytoplasmic staining (Figure 8). Antibody localisation on the cell surface became less prominent at 37°C while increase staining was observed in the cytoplasm. Cytoplasmic staining was observed to accumulate in the cell, suggesting that seeMet 2 and 13 were internalised upon binding to c-Met and accumulate within the cell.

### Temperature sensitivity of seeMet 2

**Figure 9:**
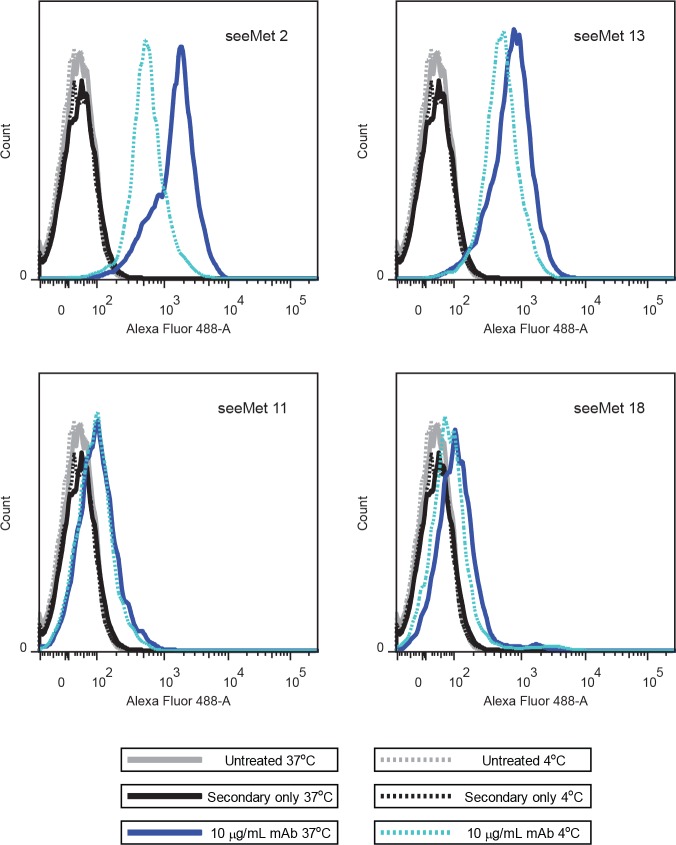
Flow cytometry analysis of seeMet monoclonal antibody binding to live SNU-5 cells at different temperatures Live SNU-5 cells were treated with respective monoclonal antibody for an hour at 4°C or 37°C. Cells were harvested. seeMet monoclonal antibody binding was detected using Alexa Fluor®488-conjugated anti-mouse secondary antibodies and cells were analysed by flow cytometry.

To determine if temperature affects the binding affinity of seeMet monoclonal antibody to native c-Met, seeMet 2, 13, 11 and 18 were incubated with live SNU-5 cells at 4°C or 37°C. Binding of monoclonal antibody to native c-Met expressed on the cell surface of live SNU-5 cells was detected using anti-mouse secondary antibody conjugated with Alexa Fluor®488 dye and analysed by flow cytometry. Antibody binding, which correlates with fluorescence intensity, was compared at the different temperatures. Interestingly, each antibody demonstrated a peak shift between different temperatures (Figure 9), indicating a decrease in antibody binding at 4°C. Among the four antibodies tested, seeMet 2 showed the most drastic peak shift. It is plausible that the reduction of antibody binding is due to the unavailability of antibody's epitope on native c-Met expressed on live SNU-5 cells. We hypothesise that c-Met is a dynamic protein at normal physiological temperature and becomes more rigid at low temperature, causing certain epitopes to be obscured.

### Alanine scan analysis of seeMet 2 main epitope

**Figure 10:**
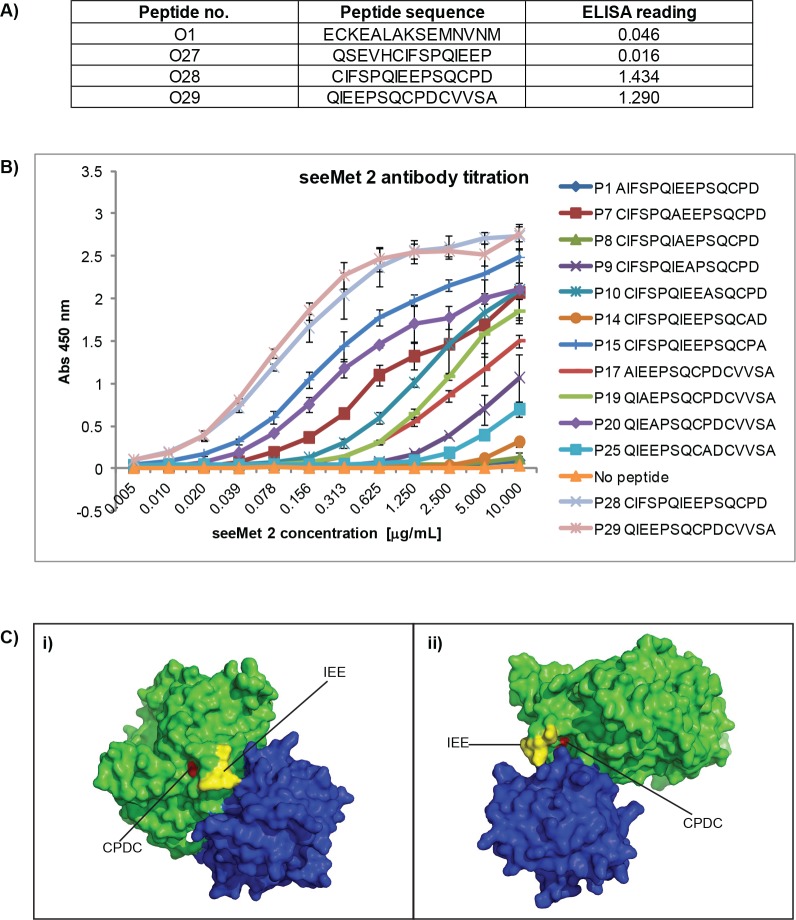
Identification of seeMet 2 main epitope A*)* Pepscan analysis of seeMet 2. seeMet 2 bound to peptides O28 and O29 with similar affinity. Peptide sequence and binding affinity are stated. Peptides O1 and O27 are used as negative controls. B*)* Titration of seeMet 2 against alanine scan peptides. Alanine scan was used to determine the main residues involved in seeMet 2 epitope. The lack of seeMet 2 binding would indicate the involvement of the substituted residues in seeMet 2-Met interaction. To determine residues that are more crucial in seeMet 2 epitope, alanine scan peptides with reduced seeMet 2 binding in the initial scanning assay were titrated against increasing concentration of seeMet 2. Titrated peptides are listed, in Table 2, in increasing binding affinity to seeMet 2. C) Mapping of seeMet main epitope on c-Met crystal structure 1SHY. c-Met is highlighted in green and HGF in blue. c-Met epitope ‘IEE’ (residue 166-168) and ‘CPDC’ (residues 172–175) are highlight in yellow and red respectively. ‘IEE’ motif lies on c-Met surface while ‘CPDC’ is hidden within c-Met. The protein complex is shown from two different viewpoints (i) and (ii). Amino acid residue number is based on c-Met protein crystal structure.

The previous pepscan analysis was designed to narrow down an antibody's epitope to be within a maximum of 15 amino acid residues. However, not all residues in the peptide may be involved in antibody-Met interaction. To further understand the decrease in antibody binding at low temperature, the epitope of seeMet 2 was mapped to greater detail. Pepscan analysis of seeMet 2 demonstrated that seeMet 2 bound to peptides O28 (CIFSPQIEEPSQCPD) and O29 (QIEEPSQCPDCVVSA) with equal affinity (Figure 10A). This indicates that the epitope of seeMet 2 is shared by both peptides. To identify critical residues in seeMet 2 interaction, variations of peptides O28 and O29, where each amino acid residue is sequentially substituted for an alanine residue, were synthesised. Peptides P1 to P16 and P17 to P31 were derived from the original peptide O28 and O29 respectively (Table 3). Utilising the same ELISA-based assay described previously, synthesised peptides were added to streptavidin-coated wells. Purified seeMet 2 was then added to each alanine-substituted peptide and antibody binding was determined by absorbance reading at 450 nM. Loss of antibody interaction to a peptide would demonstrate the importance of the substituted amino acid residue in seeMet 2-Met interaction. Peptide O27, peptide O1 and no peptide were used as negative controls. Interestingly, although the first 10 amino acids of peptide O28 overlaps with peptide O27, seeMet 2 does not bind to peptide O27. This highlights the stringent requirement of seeMet 2 binding.

**Table 2:** List of alanine scan peptides that disrupted seeMet 2 epitope

Order of binding loss	Peptide no.	Peptide sequence
1	P1	AIFSPQIEEPSQCPD
	P8	CIFSPQIAEPSQCPD
2	P14	CIFSPQIEEPSQCAD
3	P25	QIEEPSQCADCVVSA
4	P9	CIFSPQIEAPSQCPD
5	P17	AIEEPSQCPDCVVSA
6	P19	QIAEPSQCPDCVVSA
7	P20	QIEAPSQCPDCVVSA
	P7	CIFSPQAEEPSQCPD
P10	CIFSPQIEEASQCPD
8	P15	CIFSPQIEEPSQCPA
9	O28	CIFSPQIEEPSQCPD
	O29	QIEEPSQCPDCVVSA

Peptides that failed to bind seeMet 2 were titrated against seeMet 2 and listed above. The peptides are listed in increasing binding affinity to seeMet 2 which also reflects the importance of the substituted residue. For example, the substituted residues in peptide P1 and P8 are more critical in seeMet 2 epitope than in peptide P15. Peptides sequences are shown. Alanine-substituted residues are indicated in red. Peptide O28 and O29 were positive control.

In the presence of 1 mM DTT, alanine scan analysis revealed that seeMet 2 failed to bind to peptides P1, P7, P8, P9, P10, P14, P15, P17, P19, P20 and P25 when the antibody was used at 0.05 µg/mL (data not shown). The substituted amino acid residues in these peptides are thus involved in seeMet 2 epitope. Interestingly, proline residues that form kinks in protein structures were observed to be important residues in the seeMet 2 epitope. This is supported by the loss of seeMet 2 binding due to the substitution of proline by alanine residues in peptides P14, P25 and P10. Using a higher range of seeMet 2 concentration, a detailed titration of seeMet 2 binding to the alanine scan peptides was performed to further define the relative contribution of different amino acids to the interaction (Figure 10B). These extensive titrations gave very clear indications on the relative impact of the different alanine substitutions. The peptides are listed in Table 2 in the order of increasing binding affinity to seeMet 2.

seeMet 2 bound peptides P1, P8 and P14 the least, indicating that the substituted residues, ‘C’, ‘E’ and ‘P’ respectively, are most critical for seeMet 2 binding (Figure 10B and Table 2). Further analysis of seeMet 2 titration results showed that the residues ‘IEE’ and ‘CPDC’ form the main epitope. These residues are not consecutive residues. The importance of proline residues and the non-consecutive residues in seeMet 2 main epitope strongly suggests that the native structure of c-Met is important for seeMet 2 binding. This is consistent with the observed character of seeMet 2 i.e. seeMet 2 has high affinity and specificity towards native c-Met and not to denatured c-Met. It is only when a protein is in its tertiary conformation that non-consecutive resides lie side by side and come together to form a site for protein-protein interaction.

Mapping of seeMet 2 epitope onto the crystal structure of c-Met (PBD 1SHY), revealed that the main epitope fell into two distinct clusters: residues 166 to 168 ‘IEE’ and 172 to 175 ‘CPDC’ (Figure 10C). The ‘IEE’ motif, in yellow, is exposed on the surface of c-Met while the ‘CPDC’ motif, in red, is buried within c-Met. How seeMet 2 is able to bind to the cryptic ‘CPDC’ motif is not known but it is probable that this region could be more exposed by molecular dynamic fluctuations that can occur on the surface of cancer cells [12]. Computer simulation studies of seeMet 2 binding will further elucidate this cryptic epitope of seeMet 2.

**Table 3:** seeMet 2 alanine scan peptides

Original peptide	Peptide no.	Peptide sequence
O28
	P1	AIFSPQIEEPSQCPD
	P2	CAFSPQIEEPSQCPD
	P3	CIASPQIEEPSQCPD
	P4	CIFAPQIEEPSQCPD
	P5	CIFSAAQIEEPSQCPD
	P6	CIFSPAIEEPSQCPD
	P7	CIFSPQAEEPSQCPD
	P8	CIFSPQIAEPSQCPD
	P9	CIFSPQIEAPSQCPD
	P10	CIFSPQIEEASQCPD
	P11	CIFSPQIEEPAQCPD
	P12	CIFSPQIEEPSACPD
	P13	CIFSPQIEEPSQAPD
	P14	CIFSPQIEEPSQCAD
	P15	CIFSPQIEEPSQCPA
P16	AIFSPQIEEPSQAPD
O29
	P17	AIEEPSQCPDCVVSA
	P18	QAEEPSQCPDCVVSA
	P19	QIAEPSQCPDCVVSA
	P20	QIEAPSQCPDCVVSA
	P21	QIEEASQCPDCVVSA
	P22	QIEEPAQCPDCVVSA
	P23	QIEEPSACPDCVVSA
	P24	QIEEPSQAPDCVVSA
	P25	QIEEPSQCADCVVSA
	P26	QIEEPSQCPACVVSA
	P27	QIEEPSQCPDAVVSA
	P28	QIEEPSQCPDCAVSA
	P29	QIEEPSQCPDCVASA
	P30	QIEEPSQCPDCVVAA
P31	QIEEPSQAPDAVVSA

An alanine scan was used to further identify amino acid residues involved in seeMet 2 epitope. Initial studies indicate that seeMet 2 bound to peptides O28 and O29. An alanine residue, marked in red, was sequentially substituted for each amino acid residue in peptides O28 and O29. These peptides were synthesised. Peptide sequences are listed. Peptides P1 to P16 and peptides P17 to P31 are derivatives of peptides O28 and O29 respectively. All cystine residues were substituted for alanine residues in peptides P16 and P31 to determine the effect of disulphide bonds in seeMet 2 epitope.

## DISCUSSION

c-Met is a tyrosine kinase receptor involved in a wide range of biological activities such as cell proliferation, cell motility, angiogenesis and morphogenesis. Aberrant expression of c-Met correlates with tumour aggression and cancer progression. Expression of c-Met is also known to cause drug resistance towards HER2, EGFR and B-RAF treatment [13-16]. c-Met is thus an attractive target for cancer therapy.

Many groups have used various mechanisms to inhibit c-Met activation. Small molecule kinase inhibitors such as the PHA-665752, AM7 and SU11274 were extremely successful in inhibiting c-Met activation [17-19]. However, toxicity issues due to off-target effects of the small molecule inhibitors are of major concern. In addition, SU11274 was reported to be ineffective against specific c-Met mutations [20, 21]. Abounader *et al.* [22] reported the use of U1snRNA/ribozyme to downregulate the expression levels of c-Met/HGF. Although this method is novel and intriguing, at present it is not feasible to use this method for cancer treatment due to delivery issues. The U1snRNA/ribozyme has to be efficiently delivered into every tumour cell in order to be effective. The process of delivery will be a challenging problem. The use of HGF and c-Met fragments, such as NK4 and decoy-Met respectively, to compete for Met-HGF interactions has been examined. These competitive inhibitors show efficient inhibition of c-Met signaling in xenograft models [23-25]. NK4 is presently in clinical trials [26].

The success of using therapeutic monoclonal antibodies for cancer treatment is evident. Herceptin (Trastuzamab), a humanised IgG1 antibody targeted against the human epidermal growth factor receptor (HER2), is widely used for the treatment of breast cancer. Rituxan (Rituximab) targeting CD20 and Avastin (Bevaciumab) targeting vascular endothelial growth factor receptor (VEGFR), both chimeric IgG1 antibodies, have been successful in treatment of lymphomas and of colorectal and lung cancer respectively [27]. Therapeutic antibodies success in disease treatment can be attributed to their high affinity and specificity towards their target antigen. Antibody stability *in vivo* and their ability to recruit the host immune system to destroy tumour cells make this form of therapy clinically advantageous over other types of c-Met/HGF inhibitors [28]. In addition, advances in antibody engineering can improve the pharmacokinetiecs of a therapeutic antibody molecule.

There have been several reports of c-Met inhibition by anti-Met antibodies. The monovalent 5D5 antibody (MetMab) was shown to cause receptor downregulation while the monovalent DN-30 antibody was reported to inhibit c-Met signaling by causing c-Met ectodomain shedding [8, 29]. This releases the extracellular domain into the cell medium, causing the cleavage of the c-Met intracellular domain into the cell cytoplasm. Bivalent LMH 87 antibody bound to c-Met on the cell surface and resulted in receptor downregulation by cellular internalisation [11]. Presently, MetMab is in clinical trials [30]. Given the success of antibodies in cancer therapy and c-Met involvement in cancer development, the outlook for developing therapeutic antibodies against c-Met is promising.

In this study, we have developed a panel of murine monoclonal antibodies against the α-chain of human c-Met. Here, we term the antibodies: Specifically Engaging Extracellular c-Met (seeMet). We characterised the antibodies by Western blotting, immunoprecipitation, flow cytometry, epitope mapping and agonist/antagonist activity towards c-Met. The α-chain was used as immunogen as it was best expressed and most immunogenic among other c-Met fragments (full length β-chain, extracellular β-chain, extracellular domain of c-Met). Comparison with the commercial antibodies (SC-10 and AF276) has revealed the superior performance of our new antibodies in immunoblotting. seeMet 11 and 12 were the best antibodies for Western blotting as they demonstrated good affinity and specificity towards c-Met. In addition, our antibodies are able to function in immunoprecipitation and ELISA. Antibodies that have higher affinity and specificity compared to the currently available commercial antibodies, and characterised in various biochemical techniques, will be valuable tools for c-Met further studies. Finally Pozner-Moulis *et al.* [31] questioned the reliability of commercial c-Met antibodies as cancer prognosis markers. Of the five commercial c-Met antibodies examined to quantify c-Met protein levels, some antibodies had batch variation while others had non-reproducible results. This emphasises the need to develop and characterise reliable c-Met antibodies.

Despite the use of prokaryotic-expressed denatured protein as immunogen, it was still possible to obtain antibodies that recognise native c-Met protein. Antibodies such as seeMet 13, showed poor affinity and poor specificity to c-Met by Western blotting, but, along with seeMet 2, produced the highest fluorescence staining determined by flow cytometry. Clearly, seeMet 2 and 13 epitopes lies in c-Met native conformation. In SNU-5 cells, seeMet 2 and 13 do not cause caspase activation and do not cause cell death as suggested by lack of annexin V staining (data not shown). Western blot analysis of antibody-treated cells using the senescence marker and autophagy marker, p16 and LC-3 respectively, were negative for such events (data not shown). Through the binding of seeMet 2 or 13 to c-Met, the only functional effect these antibodies elicit is the reduction of SNU-5 cell division. Finally, our antibodies are non-agonistic towards c-Met in cell scatter assays.

Given the advances in antibody engineering, there are several ways antibodies may be engineered for cancer therapy and diagnosis. seeMet 2 and 13 bind to endogenous c-Met on live SNU-5 cells with high affinity and specificity. Immunofluorescence staining demonstrates that seeMet 2 and 13 are internalised into tumour cell, most likely mediated via endocytosis. Combining the technology in molecular imaging and nanotechology, c-Met antibodies may be engineered into powerful tools for *in vivo* tumour imaging. This will provide valuable information on tumour physiology which will help improve cancer diagnosis, prognosis and therapy. c-Met antibodies may also be engineered to carry toxic payloads into tumour cells and cause tumour cell killing. The very encouraging clinical results reported with Kadcyla, a Herceptin mertansine-conjugate, just obtaining FDA approval for the treatment of advanced breast cancer strongly support this idea.

So far, we have shown treatment of SNU-5 cells with 10 µg/mL of seeMet monoclonal antibody. Other groups have shown, and it is also plausible, to use higher concentrations of antibody treatment. Testing our antibodies at higher concentration might provide better insights into our antibodies’ mode of action. Interestingly, the bivalent LMH 87 antibody described by Greenall *et al.* [11] shares a similar binding epitope to seeMet 13. Like seeMet 13, LMH 87 antibody is a non-agonist, is internalised into the cell and caused cell growth inhibition. Although LMH 87 antibody does not cause c-Met ectodomain shedding, it will be intriguing to test seeMet 13 for this function.

Partial inhibition of c-Met-induced biological activities using monoclonal anti-Met antibodies has been described by Prat *et al*. [7]. The anti-Met antibody, DN-30, inhibits other c-Met-induce biological activities, but not cell motility. In the same study, another monoclonal antibody (DO-24) developed from the same immunisation as DN-30, was a full agonist capable of eliciting full c-Met activity [7]. Cao *et al.* [4] demonstrated that more than three monoclonal antibodies are required to completely inhibit c-Met binding to HGF. The molecular mechanisms of c-Met activation by HGF are still unclear. This interaction is more complex than previously thought and from these antibody studies, it is obvious that there are critical interaction sites that are responsible for eliciting various c-Met activities. In addition, the discovery of the seeMet 2 cryptic epitope suggests that c-Met is a dynamic protein and this further illustrates the lack of understanding we have on c-Met physiology. seeMet 2 may be similar to the EGFR 806 antibody, that recognises a cryptic epitope that is only expressed on tumour cells [12]. Interestingly, the 806 antibody epitope contains two cysteine residues, which are similar to the ‘CPDC’ motif in seeMet 2 epitope, forming a covalent sulfhydryl bond. Biochemical studies, computer simulation studies and antibody crystallisation studies will help to unravel this mystery and our antibodies would be the perfect tools for further c-Met studies.

## MATERIALS AND METHODS

### Cell lines and reagents

All cells were maintained at 37˚C in 5% CO_2_ humidified incubator. HaCaT, U-87MG and murine NIH3T3 cells, cultured in Dulbecco's Modified Essential Medium (DMEM) high glucose with sodium pyruvate, were generous gifts from Birgit Lane (Institute of Medical Biology (IMB), Singapore), Nick Leslie (Division of Cell Signalling and Immunology, University of Dundee) and Axel Ullrich (Institute of Medical Biology (IMB), Singapore) laboratories respectively. SNU-5 cells were purchased from Korean Cell Line Bank. T47D and SNU-5 cells were cultured in RPMI 1640 media. DMEM and RPMI were obtained from Invitrogen (U.S.A). All tissue culture medium was supplemented with 10% heat-inactivated fetal calf serum obtained from HyClone Laboratories/Thermo Scientific (U.S.A).

Recombinant human HGF (#294-HG) and anti-human HGF (#MAB294) were purchased from R&D Systems (U.S.A). SU11274, the c-Met small molecule inhibitor, was purchased from Calbiochem/Merck (Germany, #448101). CellTracker Green BODIPY dye (Invitrogen #C2102) was resuspended in DMSO, according to manufacturer's protocol.

### Cell harvest and cell lysis

Cell culture plates/dishes were rinsed with cold PBS before the cells were scraped off. Cells were collected by centrifugation. PBS was removed before quick freezing the cell pellet on dry ice. The frozen cell pellet was lysed on ice using NP40 lysis buffer (1% NP40, 150 mM NaCl, 50 mM Tris-HCL pH 8.0) containing complete protease inhibitor (Roche, United Kingdom, #11 697 498 001). Cell lysate was centrifuged at 10,000 xg and the supernatant was recovered for analysis. Protein quantification was performed using a BCA kit (Pierce/Thermo Scientific), according to the manufacturer's protocol.

### Cloning, prokaryotic expression and purification of c-Met α-chain

c-Met α-chain was first cloned from the entry vector, containing full length human c-Met cDNA (Invitrogen #IOH36570), into pCR2.1 vector (Invitrogen) using the TOPO TA cloning kit (Invitrogen). Amplification of c-Met α-chain was performed using c-Met α-chain primers (alphaF: 5'-ATATGGAGTGTAAAGAGGCACTAGC-3' and alphaR: 5'- CTATCTCTTTTTTCTCTTTTCTGTGAG-3'). All plasmid extraction, gel extraction and PCR product purification protocols were performed using Qiagen (United Kingdom) kits, according to manufacturer's protocol.

For prokaryotic expression, c-Met α-chain was subcloned from pCR2.1 vector into His-tag pET19b expression vector (Novagen/Merck) and transformed into BL21 pLysS cells (Invitrogen). Transformed cells were grown until an OD_600_ 0.4 to 0.6 was reached. Protein expression was induced by adding IPTG (isopropyl-β-D-thio-galactoside) to a final concentration of 1 mM. Cultures were allowed to grow for 3 hrs. Cells were recovered by centrifugation and resuspended in lysis buffer (0.3 M KCL, 50 mM KH_2_PO_4_, pH 8.0). Cell lysate was centrifuged at 33,000 xg. The cell pellet obtained was resuspended in lysis buffer containing 6 M urea and incubated overnight with gentle stirring. The resulting suspension was centrifuged and the supernatant obtained was used for protein purification.

Prokaryotically expressed c-Met α-chain was affinity purified on a 1 mL IMAC (immunobilised metal affinity chromatography) Bio-Scale Mini Cartridge (Bio-Rad, U.S.A), using the automated Profinia protein purification system (Bio-Rad). c-Met α-chain was eluted and used to immunise mice for antibody production.

### Mouse immunisation and hybridoma fusion

Antibody production was performed by Moravian Biotechnology (Czech Republic). Briefly, mice were immunised with purified human c-Met α-chain expressed from bacteria. Each injection contained 40 μg of purified c-Met α-chain. Mouse tail bleeds were taken to test for immunological response against c-Met α-chain. Two mice that gave the highest immune response against c-Met α-chain were sacrificed for hybridoma cell fusion. Spleen cells from mice were fused with SP2./0-Ag14 cells which are mouse immortal myeloma cells. Hybridoma cells were grown in selection media containing hypoxanthine, aminopterin and thymidine. Only hybridoma cells which have been successfully fused between a spleen cell and an immortal cell will survive and grow in the selection media.

### Antibody screening

Antibody screening was performed mainly by cell staining and Western blotting. An outline of monoclonal antibody screening is shown in Supplementary Figure 1. Hybridoma cells were screened for anti-α-chain antibody production ten days after cell fusion.

### Isotyping

Mouse monoclonal antibodies isotype were characterised using the commercially-available IsoQuick™ strips (Sigma-Aldrich). Isotyping strips were incubated in hybridoma cell supernatant for 5 mins.

### Pepscan and alanine scan

Peptides for Pepscan: Peptides that span the entire α-chain of c-Met were synthesised. A total of fifty-five peptides with consecutive, overlapping sequences were synthesised. Each peptide consists of 15 amino acid residues and overlapped the previous peptide by 10 amino acid residues.

Peptides for alanine scan: Pepscan results showed that seeMet 2 bound to peptides O28 (CIFSPQIEEPSQCPD) and O29 (QIEEPSQCPDCVVSA). Variations of peptides O28 and O29, where each amino acid residue was sequentially substituted for an alanine residue, were synthesised.

All peptides were linked to biotin by an SGSG linker sequence at the N-terminus and were synthesised by Mimotopes (Australia). Streptavidin-coated plates (Pierce/Thermo Scientific, #15520) were blocked with 3% BSA/PBS at room temperature. The peptides were dissolved in DMSO and stored according to manufacturer's recommendation. Streptavidin-coated plates were coated with each peptide (5 μg/mL) overnight at room temperature. Wells were washed before hybridoma supernatant or purified monoclonal antibody was added to the each well. HRP-conjugated anti-mouse antibody was added to detect bound monoclonal antibodies. ELISA substrate (Bio-Rad #172-1067) solution was prepared freshly according to the manufacturer's instructions and added to the wells. Colour change was monitored by eye and the reaction was stopped by adding 100 mM sulphuric acid. Absorbance was read at 450 nM in a microplate reader (SPECTRAmax PLUS^384^, Molecular Device, U.S.A). The crystal structure of c-Met extracellular domain, accession number 1SHY, was obtained from Protein Data Base (PDB). Computer imaging of the epitopes were analysed by PyMol (DeLano Scientific LLC, U.S.A) software.

### Western blotting and antibodies

The commercially-obtained SC-10 antibody (Santa Cruz, U.S.A) was raised against a peptide within the C-terminal cytoplasmic region of human c-Met and would be expected to recognise c-Met precursor (170 kD) and mature c-Met β-chain (145 kD) on Western blots. AF276 antibody (R&D systems) is a goat antibody raised against the extracellular domain of c-Met. AF276 antibody would be expected to recognise c-Met precursor, mature c-Met β-chain and mature c-Met α-chain on Western blots. Both SC-10 and AF276 antibodies were used at 1: 10 000 dilution on Western blotting. 1 µg/mL of seeMet monoclonal antibody was used for Western blotting.

HRP conjugation to seeMet antibodies was performed using EZ-link Plus Activated Peroxidase (Pierce/Thermo Scientific). HRP conjugated antibodies were used at 1 µg/mL on Western blotting.

Protein samples were mixed with 4X LDS sample buffer (Invitrogen) and 10X sample reducing agent (Invitrogen) before analysing on 4 -12% Bis-Tris gradient gels (Invitrogen) using MOPs buffer (Invitrogen). SeeBlue Plus2 protein ladder (Invitrogen) was used as molecular weight ladder. Transfer of proteins onto a nitrocellulose membrane (0.45 μm pore size, Whatman, United Kingdom) was performed using Bio-Rad wet transfer system for 2 hrs, at a constant current of 100 V. Transfer buffer contains 25 mM Tris, 192 mM glycine and 20% methanol. Blocking buffer was made up of 5% Marvel milk in PBST (1% Tween 20 in PBS). Non-specific sites on the membrane were blocked in blocking buffer before incubating with primary antibodies. The membrane was washed three times and secondary antibodies were added at 1:10 000 dilution in blocking buffer. Secondary antibodies conjugated to horseradish peroxidise (HRP) were obtained from Jacksons Laboratory/Stratech Scientific Ltd. (United Kingdom). Enhanced chemiluminescence (ECL) (Amersham/G.E. Healthcare, United Kingdom) was used for the detection of Western blots.

### Immunoprecipitation

Cells lysates were obtained by lysing cells in NP40 lysis buffer as described above. 1 µg of purified antibody was incubated overnight with cell lysate (200 μg – 500 μg of total protein in approximately 500 μL). Washed protein G beads (Sigma-Aldrich, U.S.A) were added to the cell lysate. Beads were collected by centrifugation at 18,000 xg and washed several times with NP40 lysis buffer. Bound proteins were eluted by adding 2X LDS sample buffer and heated at 100°C. Beads were removed from eluate by centrifugating at 10,000 xg. The eluate was analysed by SDS-PAGE gel.

### Flow cytometry

Flow cytometry was performed with help from Flow Cytometry Core Facility (College of Medicine, Dentistry and Nursing, University of Dundee, United Kingdom).

SNU-5 cells (1 x 10^6^ cells) were washed once in cold PBS before blocking in 1% BSA/PBS. Cells were washed twice before incubating with seeMet monoclonal antibodies. 1 μg/mL of seeMet monoclonal antibodies were used. Cells were incubated with goat anti-mouse IgG FITC-conjugated secondary antibodies (Invitrogen) and finally resuspended in 1% BSA/PBS. Flow cytometry was performed using Becton Dickinson (U.S.A) FACScan. Cell Quest (Becton Dickinson) and FlowJo (Tree Star Inc., U.S.A) software was used for data analysis.

Temperature sensitivity: 10 µg/mL of seeMet monoclonal antibody were incubated with live SNU-5 cells for 1 hr at 4°C or 37°C. Cells were harvested and washed once in cold PBS. Antibody binding to live cells was determined by Alexa Fluor®488-conjugated anti-mouse secondary antibodies (Invitrogen #A11029). Cells were resuspended in 1% BSA/PBS and analysed by flow cytometry.

### Cell scatter assay

100 - 200 HaCaT cells were seeded in 24-well plates and allowed to grow until small colonies were formed (approximately 7 days). Cells were serum-starved for 24 hrs. Cells were incubated with purified monoclonal antibodies (1 μg/mL) for 24 hrs before rinsing twice in cold PBS and fixing in ice-cold methanol. Cells were then stained in 1% crystal-violet (Sigma-Aldrich) solution. Cell staining was observed using Zeiss Axiovert 25 inverted microscope and pictures were taken using a Canon EOS 1000 D camera.

### Cell viability and caspase activation

CellTiter-Glo® luminescent cell viability assay and Caspase-Glo® 3/7 assay (Promega, U.S.A) were used to determine cell viability and caspase activation respectively. SNU-5 cells (1 x 10^5^ cells) were seeded in each 96-well and treated for 72 hrs before analysis. Cell viability and caspase activation were performed according to manufacturer's protocol. Luminescence was read using the EnVision plate reader (PerkinElmer, U.S.A). While the CellTiter-Glo® luminescent cell viability assay is generally used as a cell viability assay, it actually measures cellular ATP levels. If a cell population fails to divide over the course of an assay (as seen in the study reported here) it can appear to have reduced “viability” (lower ATP levels) without, in fact, any loss of cell viability when compared to cell populations that increase in number during the assay.

### Cell division using cell counter and CellTracker Green BODIPY dye

Cell division: Cells were seeded and treated with seeMet monoclonal antibodies for 72 hrs. Cells were harvested and counted using the automated ADAM cell counter (Digital Bio, Korea).

CellTracker Green BODIPY dye: SNU-5 cells were stained with 5 µM of CellTracker BODIPY dye for 30 mins. Stained cells were seeded and treated with seeMet monoclonal antibodies for 6 days. Cells were harvested and dye retention in live cells were analysed by flow cytometry.

### Immunofluorescence

10 µg/mL of seeMet monoclonal antibody were incubated with live SNU-5 cells for 1 hr at 4°C or 37°C. Antibody-treated cells were washed once with PBS and deposited onto polylysine-coated slides (Thermo Scientific, #5991056) using the Thermo Shandon Cytospin 3 Cytofuge (Thermo Scientific) at 800 rpm for 5 mins and fixed immediately in 4% paraformaldehyde. Cells were then blocked and permeablised in PBS containing 0.4% Triton X-100 and 5% BSA. Bound seeMet antibodies were detected using anti-mouse Alexa Fluor®488-conjugated secondary antibody. Phallodin was added together with the secondary antibodies. Cells were washed before staining with 4',6'-diamidino-2-phenylindole (DAPI) and mounted with Hydromount (National Diagnostics, U.S.A) containing 2.5% DABCO (1,4- Diazabicyclo-[2.^2^.^2^]octane) (Sigma-Aldrich) as an anti-bleaching agent. Immunofluorescence was observed using Nikon Eclipse E600 microscope.

## SUPPLEMENTARY FIGURES


