# Supplementary material for: Anti-c-Met antibodies recognising a temperature sensitive epitope, inhibit cell growth
Source: Oncotarget. 2013 Jun 29;4(7):1019–36. doi: 10.18632/oncotarget.1075 (PMC3759663; doi:10.18632/oncotarget.1075)
Supplement: Supplementary file 1 [file oncotarget-04-1019-s001.pdf]

Anti-c-Met antibodies recognising a temperature sensitive epitope, inhibit cell growth - Wong et al

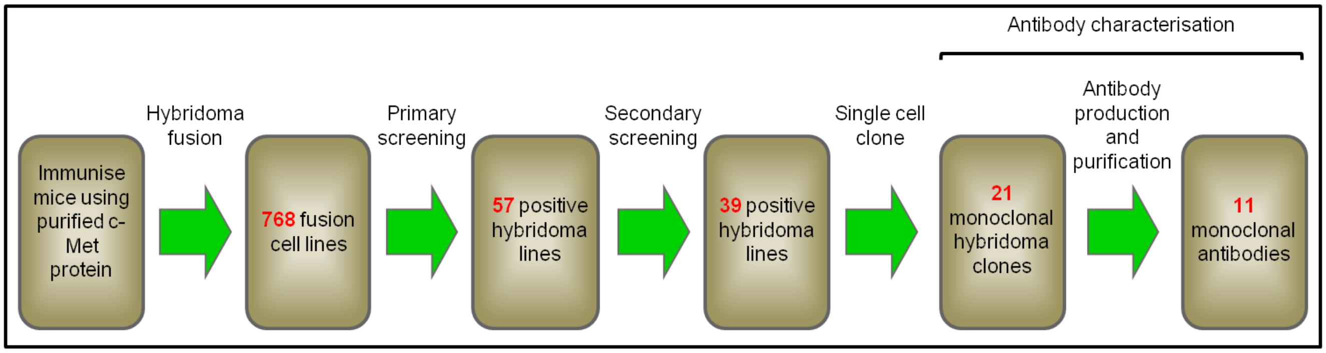

Supplementary Figure 1: Outline of monoclonal antibody screening. 768 cell lines were screened for the production of anti-α-chain antibodies after hybridoma fusion. 57 lines were found to express anti-α-chain c-Met antibodies in the primary screen. These lines were expanded and tested again for the production of anti-α-chain antibodies in the secondary screen. 39 lines were observed to stably express anti-α-chain antibodies. 21 lines were selected for single-cell cloning. These antibodies were then characterised for region of binding, isotype subclasses and functionality on Western blots. Based on these assays, 11 clones were selected for antibody production. Monoclonal antibodies were purified and characterised further.
